# FACTORS RELATED TO DELIRIUM AMONG OLDER ADULTS IN NEUROLOGICAL INTENSIVE CARE UNITS

**DOI:** 10.1093/geroni/igae098.3352

**Published:** 2024-12-31

**Authors:** Aeyoung Cho, Kyung Hee Lee, JiYeon Choi, Jung Yeon Kim

**Affiliations:** Yonsei University, Seoul, Republic of Korea; Yonsei University, Seoul, Republic of Korea; Yonsei University College of Nursing, Mo-Im Kim Nursing Research Institute, Seoul, Republic of Korea; Severance Hospital, Seoul, Republic of Korea

## Abstract

Delirium, an acute confusion states, significantly increases in intensive care unit (ICU) patients and older adults due to COVID-19 pandemic. However, patients with neurological disorders face a challenge due to the difficulty in distinguishing symptoms caused by encephalopathy; there is particularly a lack of research about older adults in ICU. We investigated the incidence of delirium and related factors among older adults in a neurological ICU during the COVID-19 pandemic. A retrospective study was conducted using electronic medical record data from 945 older adults aged 65 to 93 years admitted to the neurological ICU at a tertiary hospital in Seoul, South Korea from January 2020 to July 2023. Delirium was evaluated using the Intensive Care Delirium Screening Checklist score. Logistic regression was performed using the SPSS/WIN 26.0 program. The incidence rate of delirium was 16.9%, and 49.8% of patients were categorized as subsyndromal delirium during ICU stay. Logistic regression revealed significant factors affecting delirium among older adults in the neurological ICU, including dexmedetomidine use (OR=8.13), length of ICU stay (OR=2.89, OR=3.61 and OR=6.05 for 3-5days, 6-10days and >10days, respectively), blood urea nitrogen (BUN) levels (OR=4.55), visual impairment (OR=2.13), APACHE II score (OR=1.07) and age (OR=1.05). Accurate awareness of delirium symptoms by health professionals and identification of delirium-related factors are crucial for effective patient management. Pre-classification of delirium high-risk groups among older adults admitted to neurological ICUs is essential for providing high-quality delirium care.

